# Massive haemoptysis caused by a long‐standing foreign body in the airway

**DOI:** 10.1002/rcr2.647

**Published:** 2020-09-03

**Authors:** Hiroaki Nagano, Akiko Maeda, Takashi Kato, Ryoichi Kitamura, Wataru Higashiura

**Affiliations:** ^1^ Department of Respiratory Medicine Okinawa Chubu Hospital Okinawa Japan; ^2^ Department of Respiratory Surgery Okinawa Chubu Hospital Okinawa Japan; ^3^ Department of Radiology Okinawa Chubu Hospital Okinawa Japan

**Keywords:** Airway, foreign body, haemoptysis, inflammation, interventional radiology

## Abstract

The presence of foreign bodies (FB) in the airway is a potentially life‐threatening event. We encountered a rare case of long‐standing bronchial FB complicating with intermittent massive haemoptysis in a 42‐year‐old man. He denied any prior history of aspiration. The FB was buried deep in the bronchial epithelium and could not be removed using bronchoscopy. Bronchial angiography revealed marked dilation of the inferior lobe branch of the bronchial artery due to the FB. Bronchial artery embolization (BAE) was performed, which was followed by left lower lobectomy (LLL). The FB was keyhole‐shaped, composed of a plastic‐like material, with an appearance akin to an ancient Japanese burial mound. This case was extremely unique, since a strange FB remained in the bronchi for a long time, which caused massive haemoptysis due to the dilation of the bronchial artery.

## Graphical abstract

This case was a very unique in that a strange foreign body in the bronchi remained for a long time and massive haemoptysis was caused by dilation of the bronchial artery.

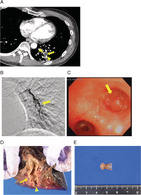

## Introduction

The aspiration of a foreign body (FB) into the airway is a potentially life‐threatening event. A long‐standing FB can induce excessive granulation tissue formation, which can make intervention more difficult. We encountered a patient with a long‐standing bronchial FB and intermittent massive haemoptysis.

## Case Report

A 42‐year‐old man from Okinawa, Japan, visited our hospital with a complaint of massive haemoptysis. He was an ex‐smoker, who had previously been in good health. He worked as a hotel receptionist. He suddenly experienced haemoptysis and visited our hospital five years ago.

At that time, chest computed tomography (CT) revealed that the left lower bronchus was obstructed by an FB. The attending physician performed flexible bronchoscopy in an attempt to retrieve the FB. However, it was lodged deeply in the bronchial epithelium and could not be removed. The haemoptysis improved spontaneously and he was discharged. Although the attending physician encouraged regular follow‐up visits at our hospital's outpatient centre, he refused due to his busy schedule.

Five years later, he developed a low‐grade fever five days before admission, in November 2019. Two days later, he expectorated around 200 mL of fresh blood (haemoptysis). Subsequently, intermittent haemoptysis occurred every day. He suddenly experienced massive haemoptysis (exceeding 500 mL) and visited our hospital.

**Figure 1:**
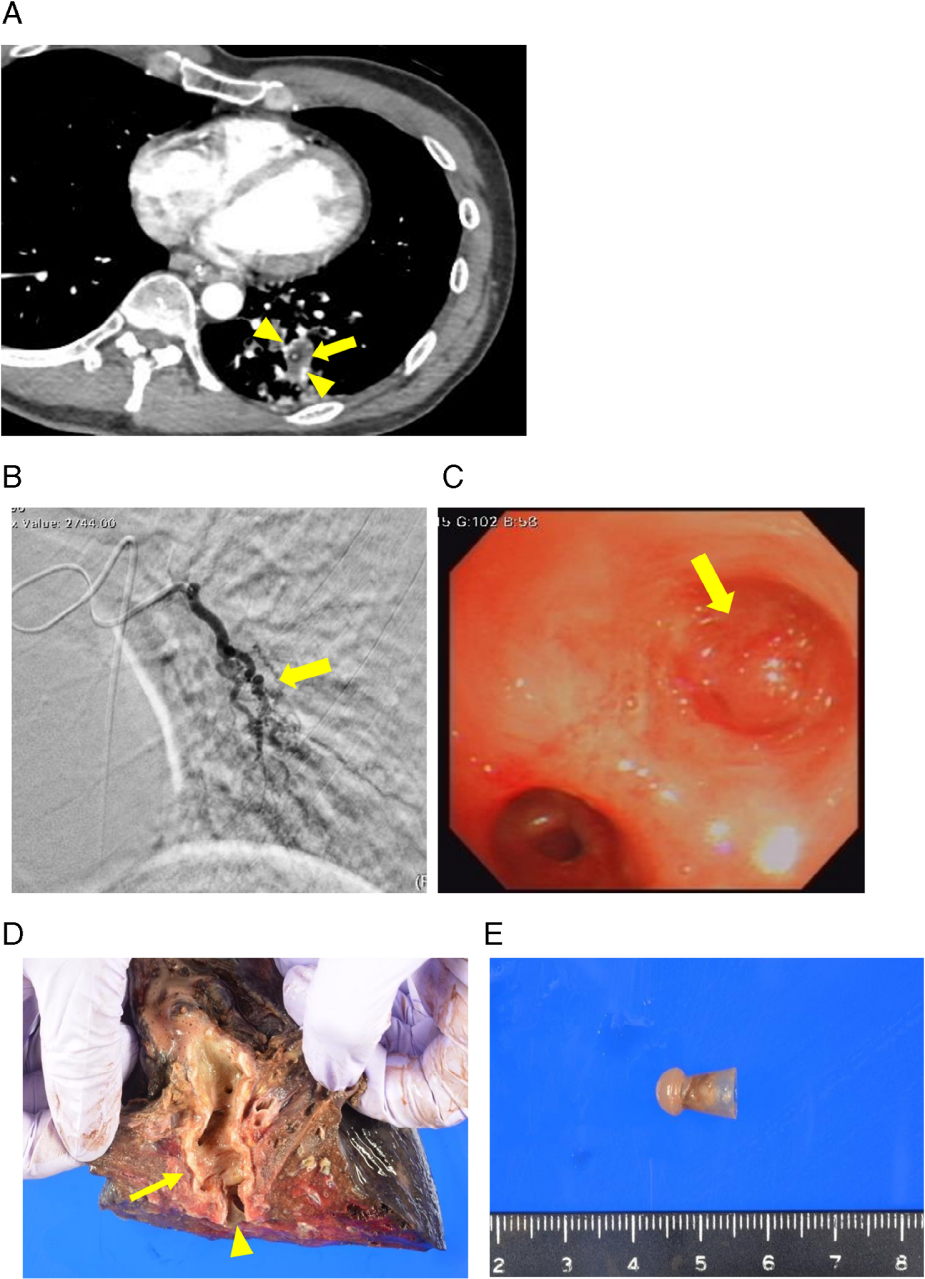
(A) Early phase of contrast‐enhanced computed tomography showing a foreign body (FB) in the lateral basal bronchus (arrow) surrounded by enhanced bronchial artery dilatation (arrowhead). (B) Bronchial angiography showing a meandering and markedly dilated (arrow) bronchial artery feeding the left lower lobe (LLL). (C) Bronchoscopy performed at the level of the left lower bronchus showing excessive granulation tissue formation and persistent oozing around the FB in the lateral basal bronchus (arrow). (D) Cross‐section of the left inferior lobar bronchi (arrow). The FB was retrieved from the lateral basal bronchus (arrowhead). (E) The shape of the FB was very unique, and its surface was translucent, with a texture similar to that of plastic or rubber.

He denied having any past history of aspiration or exposure to tuberculosis. He also denied the occurrence of chills, night sweats, productive cough, weight loss, or any recent trauma. Although he had a fever of 38°C, the other vital signs were within normal limits. Physical examination was unremarkable. The results of blood tests and urinalysis were normal. Sputum examination with Ziehl–Neelsen staining for acid‐fast bacilli and sputum cultures were all negative. Thoracic CT revealed that the left lower bronchus was still obstructed by the FB with some high‐density areas around the obstruction (Fig. 1A).

He experienced a larger amount of haemoptysis in the emergency room and was intubated to protect the airway. He was admitted to the intensive care unit. Emergency bronchial artery angiography was performed, which revealed marked dilatation of the inferior lobe branch of the bronchial artery adjacent to the FB (Fig. 1B). The radiologist performed embolization by injecting a gelatin sponge strip into the branch of the bronchial artery. The massive haemoptysis was controlled temporarily after bronchial artery embolization (BAE). We used flexible bronchoscopy to retrieve the FB again. Although we used instruments such as forceps, snare, and basket to remove the FB, granulation tissue had formed around the FB over time and the FB was buried deeply within the bronchial epithelium, which made it difficult to remove. Moreover, the FB was lodged deeply in the lateral basal bronchus epithelium (Fig. 1C).

Left lower lobectomy was performed one week after BAE owing to the risk of recurrent massive haemoptysis. Surgery was performed using the video‐assisted thoracoscopic surgery (VATS) approach with small incision. FB obstruction was not observed in the (LLL) bronchus but over the lateral basal bronchus.

Pathological examination of the resected lung lobe revealed the presence of a collapsed bronchus distal to the FB in the lower lobe with haemorrhagic changes. No neoplastic lesions were found. The FB was removed from the lateral basal bronchus of the LLL (Fig. 1D). It was very unique in shape: it was akin to a keyhole‐shaped tomb mound, that is, an ancient burial mound that is square at the front and round at the back (Fig. 1E). The FB appeared to be composed of an artificial material and not any natural substance. It was partially translucent, with a texture similar to that of plastic or rubber. It was difficult to analyse the components pathologically. The haemoptysis disappeared completely after lobectomy. He was followed up regularly in the outpatient department; recurrence of haemoptysis was not noted.

## Discussion

This study aimed to report a rare case of massive haemoptysis due to a long‐standing FB in the segmental bronchus. FB aspiration is a rare cause of massive haemoptysis [1]. We believe that late‐onset massive haemoptysis after aspiration of a plastic FB has not been reported previously.

FB aspiration is one of the most important causes of accidental deaths in childhood. Malipatil et al. reported that more than half of the FBs in the airways of children were nuts, such as peanuts [2]. Some studies have reported haemoptysis caused by FB aspiration in the respiratory tract. Misra et al. reported the case of a 42‐year‐old man with massive haemoptysis due to aspiration of a toothpick [3]. However, direct epithelial damage caused by the toothpick, which is quite sharp, seems to have caused haemoptysis. Mishra et al. reported the case of a 21‐year‐old patient with massive haemoptysis caused by the aspiration of hair [4], which led to airway epithelium inflammation resulting in haemoptysis.

In our case, bronchiectasis was not observed in the resected lung specimen. Partial dilation of the bronchial artery was observed during the interventional radiology procedure. Wu et al. reported a case with bronchiectasis around the FB [5] in which persistent respiratory infection was caused by the FB, resulting in bronchiectasis and haemoptysis. There are no reports on bronchial FB and bronchial artery dilatation, but chronic mechanical irritation or inflammation of the bronchial epithelium could have possibly induced angiogenesis.

In conclusion, this report described a non‐vegetative FB in a healthy man who had no history of aspiration. The FB remained in the bronchus for several years, causing massive haemoptysis due to bronchial artery enlargement.

### Disclosure Statement

Appropriate written informed consent was obtained for publication of this case report and accompanying images.
